# Changing trends in the global burden of mental disorders from 1990 to 2019 and predicted levels in 25 years

**DOI:** 10.1017/S2045796023000756

**Published:** 2023-11-07

**Authors:** Yang Wu, Lu Wang, Mengjun Tao, Huiru Cao, Hui Yuan, Mingquan Ye, Xingui Chen, Kai Wang, Chunyan Zhu

**Affiliations:** 1 Health Management Center, Yijishan Hospital of Wannan Medical College, Wuhu, 241001, China; 2 Department of Neurology, the First Affiliated Hospital of Anhui Medical University, Hefei, China; 3 Anhui Province Key Laboratory of Cognition and Neuropsychiatric Disorders, Hefei, China; 4 Collaborative Innovation Center for Neuropsychiatric Disorders and Mental Health, Hefei, China; 5 Department of Gastroenterology, Yijishan Hospital of Wannan Medical College, Wuhu, China; 6 School of Public Health, Wannan Medical College, Wuhu, China; 7 School of Medical Information, Wannan Medical College, Wuhu, China; 8 School of Mental Health and Psychological Sciences, Anhui Medical University, Hefei, China

**Keywords:** epidemiology, global burden of disease, incidence, mental disorder, prediction

## Abstract

**Aims:**

The burden of mental disorders is increasing worldwide, thus, affecting society and healthcare systems. This study investigated the independent influences of age, period and cohort on the global prevalence of mental disorders from 1990 to 2019; compared them by sex; and predicted the future burden of mental disorders in the next 25 years.

**Methods:**

The age-specific and sex-specific incidence of mental disorders worldwide was analysed according to the general analysis strategy used in the Global Burden of Disease Study in 2019. The incidence and mortality trends of mental disorders from 1990 to 2019 were evaluated through joinpoint regression analysis. The influences of age, period and cohort on the incidence of mental disorders were evaluated with an age–period–cohort model.

**Results:**

From 1990 to 2019, the sex-specific age-standardized incidence and disability-adjusted life years (DALY) rate decreased slightly. Joinpoint regression analysis from 1990 to 2019 indicated four turning points in the male DALY rate and five turning points in the female DALY rate. In analysis of age effects, the relative risk (RR) of incidence and the DALY rate in mental disorders in men and women generally showed an inverted U-shaped pattern with increasing age. In analysis of period effects, the incidence of mental disorders increased gradually over time, and showed a sub-peak in 2004 (RR, 1.006 for males; 95% CI, 1.000–1.012; 1.002 for women, 0.997–1.008). Analysis of cohort effects showed that the incidence and DALY rate decreased in successive birth cohorts. The incidence of mental disorders is expected to decline slightly over the next 25 years, but the number of cases is expected to increase.

**Conclusions:**

Although the age-standardized burden of mental disorders has declined in the past 30 years, the number of new cases and deaths of mental disorders worldwide has increased, and will continue to increase in the near future. Therefore, relevant policies should be used to promote the prevention and management of known risk factors and strengthen the understanding of risk profiles and incidence modes of mental disorders, to help guide future research on control and prevention strategies.

## Introduction

Mental disorders are a mental health condition with high diagnostic importance. These disorders manifest as changes in cognition, emotion and behaviour, and may be accompanied by painful experiences or dysfunction (Vargas *et al.*, 2020). Examples of mental disorders include schizophrenia, depression, bipolar disorder, anxiety disorder, eating disorder, autism spectrum disorder and conduct disorder. The pathogenic factors of mental disorders are divided into two categories: (1) biological factors, such as inheritance and infection and (2) social and psychological factors, such as stressful life events and personality characteristics. Because the aetiologies and mechanisms of these diseases remain not fully understood, these diseases can be distinguished only by a series of heterogeneous signs and symptoms that often overlap among different diseases (Levchenko *et al.*, 2020; Schmitt *et al.*, 2017). At the same time, the complex interaction among social, psychological, pathogenic and environmental factors, coupled with the difficulty in establishing an accurate model. Therefore, it is urgent to know the epidemic trend of mental disorders, which is helpful to the formulation and adjustment of disease prevention and control strategies and provides theoretical support.

Mental disorders contribute substantially to the global disease burden. They are a leading cause of disability worldwide, accounting for a significant proportion of years lived with disability. Common mental disorders, such as depression and anxiety disorders, often contribute more to the disease burden than severe mental illnesses like schizophrenia and bipolar disorder due to their higher prevalence. Notably, the prevalence of mental disorders is high, ranking in the top 10 among diseases worldwide (Charlson, et al. 2019; Collaborators 2020, pp. 1204–1222; Collaborators 2021) and the disability rate is also high (Collaborators 2020, pp. 1223–1249; Collaborators 2022). Compared with the general population, people with severe mental illness have a greater risk of death, and their life expectancy is relatively shorter (Husky *et al.*, 2020; Wilson *et al.*, 2020). Mental disorders have become an important public health problem. According to the statistics of the World Health Organization, about 1 billion people in the world suffer from mental illness. One person dies of suicide every 40 s (Arensman *et al.*, 2020). Among them, approximately 450,000 people have severe mental disorders. Thus, 7 of every 1000 families have mental disorders (Długosz and Liszka, 2021). According to a meta-analysis in 2019, the World Health Organization has estimated that the prevalence of related mental disorders, including PTSD, depressive disorder and anxiety disorder, is 22.1% among people in environments with conflict (Charlson *et al.*, 2019). As a result of the dramatic increase in stress in modern societies, the morbidity rates of mental disorders are currently likely to be higher.

In addition, mental disorders are a leading cause of disability globally. They can lead to substantial functional impairment and reduced quality of life for affected individuals. People with mental disorders often lose control of their consciousness and behaviour, and cannot participate in production and daily labour. In addition, the treatment of diseases is time consuming and costly, thus, placing many patients and their families in poverty. Furthermore, people with mental disorders have a potential risk of violent behaviour 2–4 times higher than that of individuals without mental disorders (Whiting *et al.*, 2021). In addition to many practical problems and accompanying pressures, people with mental disorders often face human rights violations, discrimination and stigma (Oexle *et al.*, 2018). People with mental disorders can have a variety of complications that aggravate their condition (Storch Jakobsen *et al.*, 2018). Among people with severe mental disorders, compared with the general population, cardiovascular morbidity and mortality are 1.5–3.0 times higher (Correll *et al.*, 2017); the incidence of diabetes is 2–3 times higher, and the incidence of overweight and obesity is 2–3 times higher (Holt, 2019; Vancampfort *et al.*, 2016). Studies have shown that offspring of parents with severe mental illness have a higher risk of death and certain physical illnesses (Ranning *et al.*, 2020). Not only people with mental disorders but also their families are affected, thus posing a heavy burden on society.

Given the limited epidemiological information on mental disorders in the global population, estimates must be updated to help guide future research on disease control and prevention strategies. This study analysed data from the Global Burden of Disease (GBD) Survey in 2019 to verify the time trends of mental disorder incidence worldwide, and to explore age, period and cohort effects by using the age–period–cohort framework. On the basis of the existing data, we aimed to predict trends in mental disorders in the next 20 years and to provide a reference for policymaking regarding intervention and treatment to reduce the global burden of mental disorders. In GBD 2019, disability-adjusted life years (DALYs) were used to measure the gap between the current health status of the population and the standard life expectancy with complete health. The data on global mental disorder incidence and disability-adjusted annual life rates from 1990 to 2019 were extracted from the official website of the GBD 2019 study.

## Materials and methods

### Data analyses

Descriptive analyses were performed on the incidence of mental disorders and DALY data by sex, age and year. To identify trends over time, we also calculated the average annual percentage changes (AAPCs) in mental disorders nationwide from 1990 to 2019. The significance of estimable functions was estimated with Wald chi-square tests. Significance was assessed at 0.05 for all analyses, and all hypothesis tests were two sided. Joint point regression analysis was used to assess trends in the disease burden of mental disorders. The analysis was performed in ‘joinpoint’ software developed by the Department of Cancer Control and Population Sciences at the National Cancer Institute. The software establishes different line segments according to the change in disease distribution over time, selects the most suitable logarithmic linear model and uses the grid search method to establish all possible connection points via an interval piecewise function. Joinpoint regression analysis can evaluate the annual percentage change (APC), AAPC and corresponding 95% confidence interval (95% CI), to provide the overall average trend of incidence. AAPC was calculated as the geometric weighted average of the various APC changes in the analysis. Finally, we evaluated whether AAPCs significantly differed from zero with *Z*-tests. Joinpoint regression program is a relatively new trend analysis method, which transforms the long-term trend into a series of meaningful segments and then describes its changing characteristics in segments. Its analysis results can provide decision-making reference for prevention and control work.

The age–period–cohort model was used to evaluate the relative risk (RR) of the population in a particular year and the accumulation of health risks since birth. The relative incidence risk [RR = Exp (*B*)] was obtained by the natural logarithmic transformation of the effect coefficient. This model enabled analysis of the independent influences of age, period and cohort on the trends in the incidence and DALY rates caused by mental disorders over time. Notably, the APC model can separate cohort effects from age and period effects in data showing trends in morbidity and mortality caused by mental disorders over time. Because ages were grouped in 5-year intervals, five consecutive years were combined into one period to achieve the same age group time span. For APC analysis, the ages were divided into continuous 5-year age groups (from 0–5 to 90–94) and successive 5-year periods (from 1990–1994 to 2015–2019). Similarly, the birth cohorts were divided into five consecutive years in intervals. The intrinsic estimation method enabled unbiased and relatively effective estimation results for age, period and cohort effects. All parameters were obtained with the freely available APC Web Tool. Age–period–cohort analysis was performed in STATA 15.0 software (StataCorp, College Station, TX, USA).

## Results

### Descriptive analysis

**Figure 1.**
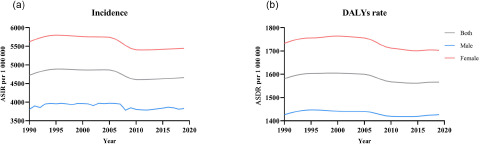
Trends in the sex-specifific age-standardized incidence (a) and DALYs rate (b) rates for mental disorders in globa from 1990 to 2019.

Figure 1 shows the age-standardized incidence rates and crude incidence rate of mental disorders from 1990 to 2019 in both sexes. In general, the age-standardized incidence of mental disorders increased in the past three decades and was higher in men than women. Over the past 30 years, the age-standardized incidence rate was higher in men than women. The curve in Fig. 1 shows that the incidence of mental disorders increased slowly from 1990 to 2005, and began to decline after 2005.

### Joinpoint regression analysis

**Figure 2.**
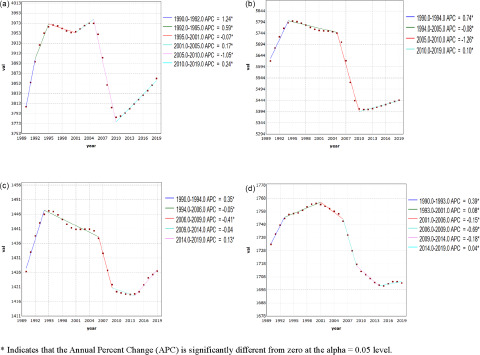
Joinpoint regression analysis of the sex-specifific age-standardized incidence and disability adjusted life years (DALYs) rate for mental disorders in global from 1990 to 2019. (a) Age-standardized incidence rate for males; (b) Age-standardized incidence rate for females; (c) Age-standardized DALYs rate for males; (d) Age-standardized DALYs rate for females and disability adjusted life years (DALYs) rate.

Table 1 and Figure 2 present the incidence of mental disorder AAPCs by sex and age group from 1990 to 2019. The incidence curves differed slightly between males and females. Specifically, the incidence from 1990 to 2010 showed an M-shaped curve in males and an inverted U-shaped curve in females. After 2010, the incidence rates in both men and women showed an increasing trend. Figure 2b and c shows the trend in the AAPC DALY rate in mental disorders globally from 1990 to 2019. Joinpoint regression analysis indicated that the male DALY rate had four turning points from 1990 to 2019, which were divided into five changing segments (AAPCs, −0.001; 95% CI, −0.024–0.021). For females, five statistically significant turning points in DALYs were identified from 1990 to 2019, which were divided into six changing segments (AAPC, −0.058; 95% CI, −0.076–0.040).

**Table 1.** Joinpoint regression analysis of the sex-specifific age-standardized incidence and disability adjusted life years (DALYs) rate for mental disorders in global from 1990 to 2019

	Males		Females	
Categories	Period	APC, % (95% CI)	Period	APC, % (95% CI)
Incidence				
	1990–1992	1.236 (1.080, 1.392)*	1990–1994	0.738 (0.666, 0.810)*
	1992–1995	0.594 (0.439, 0.749)*	1994–2005	−0.082 (−0.100, −0.065)*
	1995–2001	−0.074 (−0.109, −0.040)*	2005–2010	−1.258 (−1.329, −1.188)*
	2001–2005	0.170 (0.093, 0.274)*	2010–2019	0.099 (0.078, 0.120)*
	2005–2010	−1.049 (−1.097, −1.001)*		
	2010–2019	0.240 (0.226, 0.255)*		
AAPC, % (95% CI)		0.047 (0.024, 0.069)*		−0.117 (−0.134, −0.100)*
DALYs rates				
	1990–1994	0.347 (0.289, 0.404)*	1990–1993	0.386 (0.319, 0.452)*
	1994–2006	−0.052 (−0.065, −0.040)*	1993–2001	0.082 (0.064, 0.100)*
	2006–2009	−0.409 (−0.591, −0.228)*	2001–2006	−0.147 (−0.189, −0.105)*
	2009–2014	−0.039 (−0.096, 0.019)*	2006–2009	−0.688 (−0.82, −0.556)*
	2014–2019	0.127 (0.086, 0.168)*	2009–2014	−0.177 (−0.219, −0.135)*
			2014–2019	0.041 (0.011, 0.070)*
AAPC, % (95%CI)		−0.001 (−0.024, 0.021)		−0.058 (−0.076, −0.040)*

* *p* < 0.05, **p < 0.01, ***p < 0.001.

### Age–period–cohort analysis (APCA) with the intrinsic estimator method

**Figure 3.**
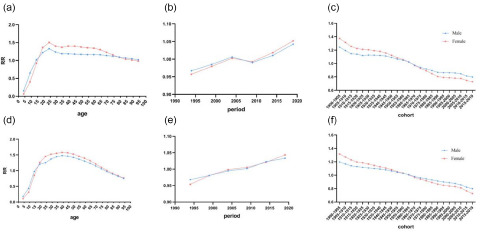
Relative risks of the incidence and disability adjusted life years (DALYs) rate due to mental disorders in global from 1990 to 2019 due to age, period, and cohort effects. (a) Age effects on incidence; (b) Period effects on incidence; (c) Cohort effects on incidence; (d) Age effects on DALYs rate; (e) Period effects on DALYs rate; (f) Cohort effects on DALYs rate.

The trends in incidence of mental disorders due to age, period and cohort effects are shown in Figure 3 and Table 2. Figure 3a shows that the incidence of mental illness in both males (RR, 1.331; 95% CI, 1.314–1.348) and females (RR, 1.504; 95% CI, 1.486–1.522) increased sharply with age and peaked at the age of 24. Subsequently, the incidence began to decrease, but rebounded slightly at the age of 39. After controlling for cycle and cohort effects, the longitudinal age effects for mental disorder incidence rates in the same birth cohort showed an inverted U-shaped pattern. As shown in Figure 3d, the DALY rates showed a rapid upward trend, peaked at the age of 39 (RR, 1.479 for males; 95% CI, 1.448–1.510; 1.579 for women, 1.549–1.611), and finally decreased slowly. In addition, the DALY rate for was slightly higher for males than women.

**Table 2.** Relative risks of the incidence and disability adjusted life years (DALYs) rate due to mental disorders in global from 1990 to 2019 due to age, period, and cohort effects

	Incidence		DALYs rates	
	Males	Females	Males	Females
Factor	RR, % (95% CI)	RR, % (95% CI)	RR, % (95% CI)	RR, % (95% CI)
**Age**				
age_4	0.152 (0.146, 0.157)***	0.065 (0.062, 0.069)***	0.177 (0.167, 0.187)***	0.107 (0.100, 0.114)***
age_9	0.651 (0.640, 0.662)***	0.404 (0.396, 0.412)***	0.437 (0.422, 0.452)***	0.313 (0.301, 0.325)***
age_14	1.017 (1.002, 1.032)	0.923 (0.910, 0.936)*	0.974 (0.949, 0.998)*	0.848 (0.827, 0.870)***
age_19	1.222 (1.206, 1.239)***	1.365 (1.348, 1.382)***	1.209 (1.181, 1.237)***	1.265 (1.237, 1.293)***
age_24	1.331 (1.314, 1.348)***	1.504 (1.486, 1.522)***	1.250 (1.222, 1.279)***	1.452 (1.422, 1.483)***
age_29	1.239 (1.223, 1.256)***	1.403 (1.387, 1.42)***	1.371 (1.342, 1.401)***	1.522 (1.491, 1.553)***
age_34	1.19 (1.174, 1.206)***	1.370 (1.354, 1.386)***	1.444 (1.414, 1.475)***	1.552 (1.522, 1.584)***
age_39	1.186 (1.171, 1.203)***	1.403 (1.388, 1.419)***	1.479 (1.448, 1.510)***	1.579 (1.549, 1.611)***
age_44	1.179 (1.163, 1.195)***	1.402 (1.386, 1.417)***	1.463 (1.433, 1.494)***	1.567 (1.537, 1.598)***
age_49	1.171 (1.156, 1.187)***	1.379 (1.364, 1.394)***	1.423 (1.394, 1.454)***	1.527 (1.498, 1.557)***
age_54	1.164 (1.149, 1.179)***	1.358 (1.343, 1.372)***	1.366 (1.337, 1.395)***	1.458 (1.430, 1.486)***
age_59	1.165 (1.150, 1.180)***	1.345 (1.331, 1.359)***	1.304 (1.277, 1.332)***	1.398 (1.372, 1.424)***
age_64	1.160 (1.146, 1.175)***	1.306 (1.293, 1.319)***	1.235 (1.210, 1.262)***	1.320 (1.296, 1.345)***
age_69	1.142 (1.128, 1.155)***	1.230 (1.218, 1.242)***	1.151 (1.127, 1.175)***	1.214 (1.192, 1.237)***
age_74	1.121 (1.107, 1.134)***	1.158 (1.146, 1.169)***	1.060 (1.037, 1.082)***	1.108 (1.087, 1.129)***
age_79	1.097 (1.084, 1.110)***	1.088 (1.077, 1.099)***	0.973 (0.952, 0.995)*	1.006 (0.987, 1.026)
age_84	1.072 (1.059, 1.086)***	1.040 (1.029, 1.051)***	0.893 (0.873, 0.914)***	0.915 (0.896, 0.934)***
age_89	1.050 (1.037, 1.063)***	1.014 (1.003, 1.025)***	0.821 (0.801, 0.841)***	0.839 (0.821, 0.858)***
age_94	1.020 (1.006, 1.035)**	0.985 (0.973, 0.997)**	0.755 (0.734, 0.776)***	0.768 (0.750, 0.787)***
**Period**				
1994	0.967 (0.961, 0.973)***	0.957 (0.952, 0.962)***	0.968 (0.958, 0.979)***	0.954 (0.944, 0.963)***
1999	0.985 (0.979, 0.991)***	0.980 (0.975, 0.985)***	0.981 (0.970, 0.991)***	0.980 (0.971, 0.990)***
2004	1.006 (1.000, 1.012)	1.002 (0.997, 1.008)	0.995 (0.984, 1.005)	0.998 (0.989, 1.008)
2009	0.990 (0.984, 0.997)	0.993 (0.988, 0.998)	1.002 (0.991, 1.013)	1.005 (0.996, 1.015)
2014	1.011 (1.004, 1.017)**	1.019 (1.013, 1.024)***	1.023 (1.012, 1.034)***	1.021 (1.011, 1.031)***
2019	1.043 (1.036, 1.049)***	1.052 (1.046, 1.057)***	1.033 (1.022, 1.045)***	1.044 (1.033, 1.054)***
**Cohort**				
1900	1.245 (1.210, 1.282)***	1.375 (1.342, 1.409)***	1.198 (1.131, 1.269)***	1.316 (1.251, 1.385)***
1905	1.195 (1.171, 1.221)***	1.316 (1.292, 1.340)***	1.169 (1.122, 1.217)***	1.272 (1.227, 1.319)***
1910	1.146 (1.127, 1.166)***	1.256 (1.237, 1.275)***	1.142 (1.105, 1.180)***	1.230 (1.194, 1.267)***
1915	1.139 (1.122, 1.156)***	1.224 (1.208, 1.240)***	1.130 (1.098, 1.162)***	1.197 (1.167, 1.228)***
1920	1.120 (1.105, 1.136)***	1.213 (1.198, 1.227)***	1.118 (1.090, 1.147)***	1.186 (1.160, 1.214)***
1925	1.126 (1.112, 1.140)***	1.207 (1.193, 1.220)***	1.108 (1.083, 1.133)***	1.167 (1.143, 1.191)***
1930	1.124 (1.110, 1.139)***	1.193 (1.180, 1.206)***	1.099 (1.075, 1.125)***	1.146 (1.123, 1.170)***
1935	1.122 (1.107, 1.137)***	1.183 (1.170, 1.197)***	1.090 (1.066, 1.116)***	1.127 (1.104, 1.151)***
1940	1.112 (1.098, 1.127)***	1.157 (1.144, 1.171)***	1.080 (1.056, 1.105)***	1.106 (1.083, 1.130)***
1945	1.090 (1.075, 1.105)***	1.122 (1.109, 1.136)***	1.064 (1.040, 1.089)***	1.082 (1.059, 1.106)***
1950	1.067 (1.052, 1.082)***	1.086 (1.073, 1.100)***	1.045 (1.020, 1.069)***	1.054 (1.032, 1.077)***
1955	1.050 (1.035, 1.065)***	1.058 (1.045, 1.071)***	1.029 (1.005, 1.053)*	1.032 (1.010, 1.055)**
1960	1.024 (1.009, 1.039)**	1.024 (1.011, 1.037)***	1.008 (0.985, 1.032)	1.005 (0.983, 1.027)
1965	0.975 (0.961, 0.990)**	0.970 (0.957, 0.983)***	0.980 (0.957, 1.003)	0.968 (0.947, 0.990)**
1970	0.942 (0.928, 0.956)***	0.930 (0.918, 0.943)***	0.961 (0.938, 0.984)	0.942 (0.921, 0.963)***
1975	0.92 (0.907, 0.934)***	0.892 (0.881, 0.904)***	0.947 (0.925, 0.97)***	0.918 (0.897, 0.939)***
1980	0.896 (0.883, 0.909)***	0.850 (0.838, 0.861)***	0.931 (0.909, 0.953)***	0.892 (0.871, 0.913)***
1985	0.868 (0.856, 0.881)***	0.805 (0.793, 0.816)***	0.914 (0.891, 0.937)***	0.867 (0.846, 0.889)***
1990	0.864 (0.851, 0.877)***	0.792 (0.780, 0.804)***	0.900 (0.877, 0.924)***	0.851 (0.828, 0.874)***
1995	0.863 (0.849, 0.878)***	0.787 (0.774, 0.801)***	0.889 (0.863, 0.916)***	0.838 (0.814, 0.864)***
2000	0.860 (0.844, 0.878)***	0.782 (0.767, 0.798)***	0.877 (0.848, 0.908)***	0.831 (0.802, 0.861)***
2005	0.848 (0.828, 0.869)***	0.778 (0.758, 0.798)***	0.857 (0.819, 0.896)***	0.812 (0.774, 0.851)***
2010	0.814 (0.784, 0.844)***	0.748 (0.717, 0.781)***	0.822 (0.765, 0.882)***	0.766 (0.706, 0.831)***
2015	0.798 (0.729, 0.875)***	0.728 (0.643, 0.824)***	0.799 (0.692, 0.924)**	0.727 (0.608, 0.868)***
Deviance	0.676	1.969	0.082	0.189
AIC	11.334	12.409	9.910	10.136
BIC	−276.084	−188.171	−316.465	−309.208

RR, relative risk; CI, confidence interval; AIC, Akaike Information Criterion; BIC, Bayesian Information Criterion.

* *p* < 0.05,

** *p* < 0.01,

*** *p* < 0.001.

In general, both sexes had similar age-standardized incidence rates and DALY rates of period effects. Regarding period effects, the incidence of mental disorders increased gradually over time, but a sub-peak was observed in 2004 (Figure 3b) (RR, 1.006 for males; 95% CI, 1.000–1.012; 1.002 for women, 0.997–1.008). For the DALY rates, the incidence rates were slightly higher in males than females, except from 2004 to 2009 (Figure 3e).

Figure 3c and f shows the estimated cohort effects by sex. In terms of cohort effects, the RR of mental disorders continued to decrease. Specifically, the incidence rate in males and women showed a turning point in 1960 (RR, 1.024 for males; 95% CI, 1.009–1.039; 1.024 for women, 1.011–1.037). Before 1960, the incidence rate in women was slightly higher than that in males; subsequently, an opposite trend was observed. Regardless of the age-standardized incidence rates or the DALY rates, a similar change trend was observed.

### Predictions of mental disorder incidence

**Figure 4.**
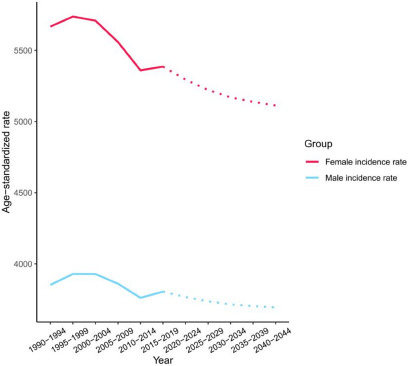
Trends in mental disorders incidence by sex in global: observed (solid lines) and predicted rates (dashed lines).

The global incidence of mental illness among males and women was predicted to show a similar trend of continuous decline. Although the trend was similar between females and males, it remained higher. The incidence is expected to be relatively stable after 2020, owing to increasing emphasis on mental disorders and a series of effective treatment measures, as shown in Figure 4. Although the incidence of mental disorders was estimated to have declined, the number of affected individuals worldwide is expected to continue to increase over the next 20 years, because of population growth and increasing societal pressures. These findings emphasize the importance of preventing and treating mental disorders.

## Discussion

To the best of our knowledge, this is the first study based on the GBD 2019 database to use the APC framework to explore global trends in mental disorder incidence and DALY rates from 1990 to 2019. Furthermore, we predicted the incidence trend of mental disorders in the next 20 years.

From 2005 to 2010, the curve changes of the incidence of mental illness and DALY rate decreased significantly. It is attributed to many factors, including changes in social attitudes and progress in health care and public health initiatives. Since 2005, the field of global mental health has continued to evolve, with increased recognition of the importance of mental health as a global public health priority. Efforts to reduce mental health disparities, provide culturally sensitive care, and expand access to mental health services have expanded, reflecting a growing commitment to addressing mental health needs on a global scale. Many countries and organizations began to pay attention to mental health problems, and took measures to improve the availability of mental health services, reduce the social stigma of mental health problems, raise awareness of mental health problems, and provide more support and treatment options. These efforts help to improve mental health around the world. The incidence and DALY rates of mental disorders differed between males and women from 1990 to 2019. The incidence increased significantly in males and decreased significantly in women, with AAPCs of 0.047 and −0.108, respectively. The DALY rates differed but decreased in both males and women. Only the female DALY rates were significant, with AAPCs of −0.001 and −0.058, respectively. These results indicated that societal attitudes and norms around mental health have been evolving. Men may be more willing to seek help or be diagnosed with mental health conditions than in the past due to reduced stigma, which could lead to higher incidence rates among men. Conversely, the decreasing incidence among women might be linked to better mental health awareness and access to treatment, resulting in improved overall mental well-being. If women have better access to mental health services or are more likely to seek help when needed, this could lead to lower DALY rates (Maki *et al.*, 2018). Despite the decreased incidence in females, the incidence remained higher than that in males. Therefore, more women than men worldwide are affected by mental disorders. On the one hand, women are often responsible for maintaining normal family life, and they bear more family and social pressures than men. Because of their lower income and savings than those of men, women are in a more disadvantageous position in terms of economic and social security.

Age is an important demographic risk factor affecting the incidence of mental disorders and disability-adjusted life span. In our research, with increasing age, the incidence of mental disorders and the annual rate of disability-adjusted life showed an inverted U-shaped change, in agreement with findings from previous studies (Charlson *et al.*, 2018). The age effect analysis indicated that the incidence of mental disorders in men and women did not increase with age, but peaked in adulthood, then declined gradually. The main reason for this finding may be that people in this stage must take on more social responsibilities and are exposed to more pressures, thus tending to lead to psychological problems. In this study, the period effect of the incidence of mental disorders generally showed an upward trend, whereas the cohort effect generally showed a downward trend. Notably, from 2004 to 2009, the incidence showed a short-term downward trend. The cohort effect represents the difference between groups born in the same year or the same age. The cohort effect generally showed a downward trend by birth year, possibly because younger birth cohorts with better education may have greater awareness of health and disease prevention than older birth cohorts (Cohen and Syme, 2013). In addition, continued government attention and investment may partly explain the continued decline in cohort risk of the incidence of mental disorders. The results of this research show that mental disorders pose a large burden.

This study predicted that the incidence of mental disorders in men and women will continue to rise in the next 30 years, and the incidence of mental disorders in women will continue to be higher than that in men. After the Coronavirus Disease 2019 (COVID-19) outbreak in 2020, the number of people with mental health problems has significantly increased, particularly among women and young people (Daly *et al.*, 2022). The COVID-19 pandemic has severely affected mental health, and attention must be paid to people whose psychological problems have not been solved. While the construction of social psychological service system progresses steadily, attention must be paid to the important role of mental health in public health, and psychological rescue should be incorporated into early warning mechanisms for disaster relief in major public health emergencies. The passive participation of psychological crisis intervention should transition to active intervention after problems occur, to ensure that affected individuals are supervised by competent professionals, and to increase their access to mental health services to help them cope with the psychological distress associated with COVID-19, thus decreasing the possible psychological harm caused by the pandemic.

In addition, we compared the predicted results with real-world data, to gain a clearer understanding of the effects of major public health emergencies on the mental health of the entire population and the need for psychological intervention.

Although this study used the APC framework to illustrate the potential effects of long-term trends in the incidence of mental disorders, it may provide additional information to help understand the burden of mental disorders. However, some study limitations should be noted. On the one hand, because the mortality rate in mental disorders is unclear, this rate was not specifically analysed in our research. Mental disorders include many types of diseases, and each disease was not specifically analysed in the research. Future research could further analyse trends in the onset of various types of mental disorders.

## Conclusion

In summary, between 1990 and 2019, the incidence of mental disorders in males and females worldwide varied dynamically. Overall, the global incidence rate increased significantly in males and decreased significantly in females. In addition, according to APC analysis, the age effects showed an inverted U-shaped curve. That is, the adult population had the highest incidence. The cycle effects demonstrated that the risk of mental disorders increased with time. Strengthening understanding of the risk profiles and the incidence patterns of mental disorders will aid in early identification of individuals at risk of mental disorders, thus supporting timely initiation of intervention measures to effectively alleviate the burden of mental disorders. Simultaneously, considering the adverse effects of the COVID-19 pandemic at the psychological level, more attention must be paid to vulnerable groups, and policies must be formulated to promote mental health.

## Data Availability

Data on the global incidence of mental disorders and disability-adjusted life rates from 1990 to 2019 were obtained from the official website of the Global Burden of Disease Study 2019 (GBD 2019) (http://ghdx.healthdata.org/gbd results-tool).
